# Biocompatibility studies of polyurethane electrospun membranes based on arginine as chain extender

**DOI:** 10.1007/s10856-021-06581-z

**Published:** 2021-08-20

**Authors:** Georgina Alejandra Venegas-Cervera, Andrés Iván Oliva, Alejandro Avila-Ortega, José Manuel Cervantes-Uc, Leydi Maribel Carrillo-Cocom, Juan Antonio Juarez-Moreno

**Affiliations:** 1 Facultad de Ingeniería Química, Periférico Norte Kilómetro 33.5, Universidad Autónoma de Yucatán, Col. Chuburná de Hidalgo Inn, C.P. 97203 Mérida Yucatán, México; 2 Centro de Investigación y de Estudios Avanzados del IPN, Unidad Mérida, Depto. de Física Aplicada, Km. 6 Antigua Carretera a Progreso A.P. 73, Cordemex, C.P. 97310 Mérida Yucatán, México; 3 Centro de Investigación Científica de Yucatán, A.C., Unidad de Materiales, Calle 43 No. 130 x32y 34, Col. Chuburná de Hidalgo, C.P. 97205 Mérida Yucatán, México

## Abstract

Electrospun polymers are an example of multi-functional biomaterials that improve the material-cellular interaction and aimed at enhancing wound healing. The main objective of this work is to fabricate electrospun polyurethane membranes using arginine as chain extender (PUUR) in order to test the fibroblasts affinity and adhesion on the material and the polymer toxicity. Polyurethane membranes were prepared in two steps: (i) the polyurethane synthesis, and ii) the electrospinning process. The membranes were characterized by scanning electron microscopy (SEM), Fourier transforms infrared spectroscopy, gel permeation chromatography, and differential scanning calorimetry techniques. The evaluation of PUUR as a scaffolding biomaterial for growing and developing of cells on the material was realized by LIVE/DEAD staining. The results show that the fluorescent surface area of human fibroblasts (hFB), was greater in control dense membranes made from Tecoflex than in electrospun and dense PUUR. From SEM analysis, the electrospun membranes show relatively uniform attachment of cells with a well-spread shape, while Tecoflex dense membranes show a non-proliferating round shape, which is attributed to the fiber’s structure in electrospun membranes. The cell morphology and the cell attachment assay results reveal the well spreading of hFB cells on the surface of electrospun PUUR membranes which indicates a good response related to cell adhesion.

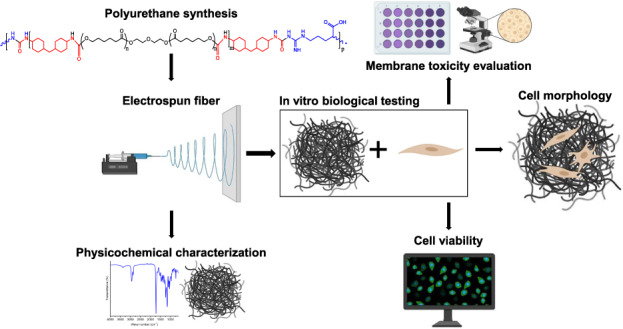

## Introduction

Polyurethanes (PUs) are synthetic polymers used for medical applications due to their semipermeability [1], good mechanical properties, easy processing, and biocompatibility [2]. These characteristics have permitted to use the PUs in permanent medical devices, vascular scaffolds [3], and cutaneous scaffolds [4]. Because of skin damage that unprotects the body against infections and sudden changes in the environment [5], cutaneous scaffolds assist in tissue reparation and regeneration when injury occurs or when a decrease in the patient’s ability to heal occurs [1]. Despite the biocompatibility of PUs, the main disadvantage is their limited cell recognition and, therefore, low levels of cell adhesion and proliferation [6], which affects its use as a skin scaffold, since it does not improve the healing time of the wound.

For improving the biological affinity of the material, different methodologies such as the synthetic and natural polymers combination [1], the insertion of biomolecules as peptides and/or amino acids on the surface [7] or in the polymer backbone (as a chain extender) [6], and the electrospinning process for converting the polymer in nanofibers, have been reported [8]. In particular, incorporation of amino acids as polyurethane chain extenders can increase the adhesion and proliferation response [7, 9].

The choice of the type of amino acid to integrate in PUs plays an important role, due to the relationship between the chemical composition of the scaffold (functional groups) and the cellular affinity, where the former modifies the wettability of the material according to the polarity [10]. Cell adhesion is influenced by both the wettability of the scaffold surface and by the cell-surface chemical interactions; however, a moderate wettability is preferable since hFB cell growth is reduced on super hydrophobic and hydrophilic surfaces [11].

On the other hand, the electrospinning technique is used to manufacture electrospun polymers [12], which promote cell-material interactions, because fibers increase the surface area for adhesion [13]. Fibrous PU structures have shown a superior effect on fibroblast cell viability over dense PU membranes [4]. Depending on the electrospinning parameters, different morphology, porosity, pore diameter, and diameter of fibers can be obtained, being the latter the most studied, having an impact on the cellular response [14].

For cell adhesion studies, the cell type is a considerable aspect, since two cell types can have different adhesion results on the same evaluated scaffold [10, 15]. Fibroblast cells are the ones responsible for regenerating the extracellular matrix in the proliferative stage of wound healing [8], so it is a cell line that is regularly used for the study of cutaneous scaffolds [16]. Likewise, it has been observed that the affinity of fibroblast cells is favorable in scaffolds with amino groups [17], or positive charges on the surface [15]. Therefore, the amino acid arginine was chosen as chain extender due to the amine groups in its structure.

The synthesized polyurethane will be studied by in vitro assays with fibroblast cells and compared with polyurethane (PUUE) extended with ethylenediamine (ED) and Tecoflex. The ED was compared with arginine as a chain extender in terms of functional groups, where both are anchored to the polymer backbone chain by urea bonds [3]. In addition, ED presents shorter length and can improve elasticity, elongation, and strength in biological applications [18, 19]. There were already some commercial polyurethane products that used ED in artificial ligament reconstruction [20], bone regeneration membrane [18], and cardiac assist devices [21]. Regarding Tecoflex, it was also used as a control in some experiments due to being a commercial polyurethane of medical-grade [3, 6].

In this work, the synthesis, physicochemical characterization, and in vitro biological tests of polyurethanes (PUUR) based on polycaprolactone diol (PCL-diol), 4,4’-dicyclohexylmethane diisocyanate (HMDI), and arginine amino acid, as a chain extender, were reported. In the same way, in vitro biological tests were carried out for PUUR, Tecoflex (commercially available medical grade polyurethane) and a polyurethane (PUUE) extended with ethylenediamine (chain extender non-amino acid) with comparative purposes. The main contribution of this work is to study the effect of amino acid incorporation and membrane topography on the adhesion of human fibroblasts (hFB). In addition, as far as we know, this is the first time that electrospun was used for preparing PUUR fibrous membranes.

## Materials and methods

### Materials

All chemicals and reagents were obtained from Sigma-Aldrich, excepting the arginine, tetrahydrofuran (THF), and Tecoflex SG80A which were supplied by Hycel, Fermont, and Lubrizol, respectively.

The used cells were human fibroblasts (hFB) obtained by the Instituto Tecnológico y de Estudios Superiores de Monterrey (ITESM), Mexico.

### Polyurethane synthesis

The synthesis of polyurethane was carried out in two stages by reacting polycaprolactone diol (PCL-diol) (2000 g mol^−1^), 4,4’-dicyclohexylmethane diisocyanate (HMDI), and arginine or ethylenediamine (ED) as chain extender, in a molar ratio 1:2.05:1, respectively [3]. In the first stage, PCL-diol reacted with HDMI at 60 °C for 4 h using dimethylformamide (DMF) as a solvent and stannous octoate 0.3% w/w as a catalyst, under nitrogen atmosphere. In the second stage, the chain extender was dissolved in DMF, added to the prepolymer, and stirred for 2 h. Upon completion, the polymer was precipitated in distilled water for 12 h. Finally, the polymer was dried in vacuum pressure at 60 °C for three days. An additional polyurethane was synthesized with ED as the non-amino acid-based chain extender under similar conditions with PCL-diol as the soft segments and HDMI as the diisocyanate.

### Manufacture of the electrospun membranes

PUUR or PUUE were dissolved in a DMF/THF mixture (3:7 v/v). The polymer solution was introduced in a plastic syringe (10 ml) with a steel needle and internal diameter of 500 µm. Electrospinning was carried out with TL-01 equipment (Tong Li Tech Co. Ltd, PR China) and a B. Braun Perfusor Space microinjection pump (B. Braun Melsungen AG). The electrospinning conditions for the PUUR, PUUE, and Tecoflex membranes preparation are described in Table 1. Difficulties regarding the processibility of the polyurethane elastomers have limited the membrane uniformity and reproducibility, therefore different electrospinning parameters in membranes preparation were used. The distance between the tip and the collector was 13 cm. The membranes were exhaustively dried under vacuum for 24 h.

**Table 1:** Electrospinning parameters to prepare different membranes

	Polymer concentration (%)	Applied voltage (kV)	Flow rate (ml h^−1^)
Tecoflex	9	20	1.0
PUUE	20	10	1.2
PUUR			
A	20	18	1.2
B	15	20	0.8
C			1.6

### Physicochemical characterization

#### Gel permeation chromatography

Gel permeation chromatography was carried out in an Agilent 1100 equipment coupled to two simultaneously Phenogel columns with nominal pore size of 10^−3^ and 10^−5^ Å. THF was used as eluent, with a flow rate of 1 ml/min at 25 °C. Polymer solutions were prepared by dissolving 10 mg of PUUR or PCL-diol in 1 ml of THF and filtering through a 0.45 µm-filter. One-hundred-µl of each solution was injected into the chromatograph. A calibration curve was elaborated with monodisperse polystyrene standards (10 mg/ml) from 1660 to 170800 g mol^−1^ for calculating the molar masses of PCL-diol and PUUR.

#### Fourier transform infrared spectroscopy

Structural properties of PUUR and PCL-diol were studied by Fourier Transform Infrared (FTIR) Spectroscopy. 10 mg of PUUR or dry PCL-diol was dissolved in 1 ml of THF. An aliquot of the solution was deposited on KBr pellets to form a polymer film on the pellets upon THF evaporation. Spectra were recorded at room temperature in a 4000 to 650 cm^−1^ range, resolution of 4 cm^−1,^ and 100 scans [6] using a Nicolet 380 FTIR spectrometer (Thermo Electron Corporation).

#### Differential scanning calorimetry

Differential scanning calorimetry (DSC) analysis of the thermal behavior of the first and second runs for the PUUR, PCL-diol, and HMDI: PCL-diol (1:1 mol/mol) samples were carried out in a Perkin-Elmer DSC-6 under nitrogen atmosphere. Specimen with weight ranging between 8–10 mg (precision of 0.01 mg), were sealed in Perkin-Elmer aluminum pans and scanned in a range of temperature from 10 to 100 °C and heating rate of 10 °C/min.

#### Scanning electron microscopy

The morphology of the electrospun membranes was studied by scanning electron microscopy (SEM) in a JEOL JSM-7500F equipment. The dried samples were metallized in a Quorum Q150R ES by depositing a thin layer of gold/palladium (60/40 at%) on the membranes for 40 s for electrical conduction. The SEM images obtained at 10,000X and their further processing for obtaining the fiber and pore diameters were done by the free-software ImageJ by taking 50 fibers.

#### Water contact angle

The contact angle on the membrane surfaces was measured with a RaméHart Model 250 optical goniometer equipped with a micro-syringe accessory using the sessile drop method. The membrane was located under the syringe and a 5 µl drop of deionized water was dropped. For each membrane, the reported contact angle represents the mean value of five measurements done on different sites of the sample surface.

### In vitro biological testing of electrospun and dense membranes

#### Sterilization and washing of the membranes

Membranes were cut in ⁓3 mm diameter circles and sterilized on both sides by ultraviolet (UV) light in a laminar flow hood for 20 min. Subsequently, they were washed with sterile distilled water and sonicated for 1 h in cold water. The washing process was repeated each 24 h for three days. For membranes care, ones were located in the 24-well plates (Sarsted) and exposed again at UV light for 20 min. Finally, phosphate-buffered saline (PBS) supplemented with 1% antibiotic and antifungal (100000 U/ml penicillin, 100 µg/ml streptomycin, and 25 µg/ml amphotericin B) (Gibco) was added to the membranes and removed after 24 h. Then, the samples were rinsed with sterile PBS for further use [3].

#### Cell culture

The human fibroblast (hFB) cells grown in 75 cm^2^ culture flasks with Dulbecco’s modified Eagle medium (DMEM) and Ham’s F-12 (Caisson Labs), 1:1 mixture medium, supplemented with 10% (v/v) fetal bovine serum (Biowest) and 1% antibiotic and antifungal [22], were incubated at 37 °C in a 5% CO_2_ atmosphere. Upon reaching cell confluence, the cells were detached with 5 ml of 0.5% trypsin (Caisson Labs). After 5 min, the process stops with a complete medium and the cells were centrifuged and resuspended in fresh culture medium. Cell concentration was determined with the trypan blue assay, 50 μl of resuspended cells were stained with 20 μl of dye solution and counting in the Neubauer chamber [23]. The tests were carried out in 24-well plates, changing the medium every two days. Incubation was carried out under the mentioned conditions.

#### Membrane toxicity evaluation

The toxicity of the membranes was determined by two methodologies: by microscopic observation of the cultured cells in contact with electrospun membranes, and by determining the cell viability respect to the cells exposed to leach from electrospun.

Particularly, in the first methodology, the cells were seeded at 1 × 10^5^ cells/well and incubated at 37 °C. Two groups of membranes were evaluated: with the washing process (Section 2.4.1), and without washing. After 24 h, growth of cells at the periphery of the membranes was observed through a LEITZ Labovert FS inverted light microscope at 10x. The presence and morphology of the cells around the membranes were compared with cells incubated without membrane (control) [24].

The second methodology of colorimetric assays with 3-(4,5-dimethylthiazol-2-yl)-2,5-diphenyltetrazolium bromide (MTT) allows determining the relative cell viability. The leachates of the PUUR, PUUE, and Tecoflex membranes were obtained by placing the membranes in sterile culture medium for 48 h (100 mg of polymer for 1 ml of medium). Subsequently, the leachates were added to hFB cells previously incubated for 24 h with a concentration of 1 × 10^5^ cells/well [3]. After 24 h, the medium was removed from the wells and 200 µl of fresh medium and 20 µl of MTT (5 mg/ml) were added; the plates were incubated for 4 h. Finally, the supernatant was carefully removed from each well, the formazan crystals were dissolved in 300 µl of DMSO [25], and the absorbance at 570 nm-wavelength was obtained by using the Thermo Fisher Scientific Genesys 20 (4001) spectrophotometer [26]. The percentage of cell viability (% CV) was calculated from the relation:

1 $$\% {{{{{\mathrm{CV}}}}}} = \left[ {\frac{{{{{{{\mathrm{Absorbance}}}}}}\,{{{{{\mathrm{of}}}}}}\,{{{{{\mathrm{cells}}}}}}\,{{{{{\mathrm{incubated}}}}}}\,{{{{{\mathrm{with}}}}}}\,{{{{{\mathrm{leachates}}}}}}}}{{{{{{{\mathrm{Absorbance}}}}}}\,{{{{{\mathrm{of}}}}}}\,{{{{{\mathrm{cells}}}}}}\,{{{{{\mathrm{incubated}}}}}}\,{{{{{\mathrm{in}}}}}}\,{{{{{\mathrm{normal}}}}}}\,{{{{{\mathrm{medium}}}}}}\,{{{{{\mathrm{only}}}}}}}} \times 100} \right]$$

#### Cell adhesion

Cell adhesion was determined using the LIVE/DEAD Invitrogen™ colorimetric assay. For this, hFB cells with 1 × 10^5^ cells/well concentration and volume of 600 µl of culture medium were incubated in plates containing electrospun (PUUR, PUUE, and Tecoflex) and dense (PUUR D, PUUE D, and Tecoflex D) membranes. After 48 h of seeding, the culture medium was removed, and the adhered cells were washed with PBS. Subsequently, a mixture of calcein AM and ethidium homodimer-1 prepared in PBS, according to the supplier’s, was added to the wells and incubated for 40 min in dark and at room temperature. The samples were observed in a LEICA MZ FLIII fluorescence stereomicroscope equipped with GFP3 and DSRED filters, and micrographs were taken at 4x to show the adherence of cells on the surface of the membranes. Green fluorescence for live cells and red fluorescence for dead cells were observed. Subsequently, the obtained micrographs were processed with the ImageJ software, and the fluorescent surface area corresponding to living cells was calculated.

#### Cells morphology

Electrospun and dense membranes of PUUR, PUUE, and Tecoflex were inoculated with 1 × 10^5^ cells/well and incubated for 24 h. After that, the culture medium was removed, and the samples washed with PBS. 2.5% glutaraldehyde, prepared in 0.1 M sodium cacodylate (pH 7.2), was added to fix the cells. After 1 h, the excess glutaraldehyde was removed and washed again with PBS. Dehydration of the samples was carried out by immersing them in increased concentrations of ethanol (30, 50, 70, 90, and 100% v/v) for 10 min for each concentration. When the ethanol was removed, hexamethyldisilazane was added for 3 min for drying [27]. Then, the dried samples were analyzed in a scanning electron microscope to obtain micrographs at 1000x.

## Results

### Polymer characterization

The molecular weights distribution of PUUR and PCL-diol precursor were calculated with gel permeation chromatography (GPC). The GPC results are listed in Table 2 and show that PUUR has the higher Mw distribution due to chain extension reaction. Also, PUUR presented a polydispersity (Pi) (Mw/Mn <2.06), which is typical of segmented polyurethanes [28].

**Table 2:** Average molecular masses for polymer samples

	*Mw*	*Mn*	*Pi*
PCL-diol	5.25 × 10^3^	3.64 × 10^3^	1.44
PUUR	4.11 × 10^4^	1.98 × 10^4^	2.06

*Pi* weight-average molecular weight/number-average molecular weight, or Mw/Mn)

**Fig. 1:**
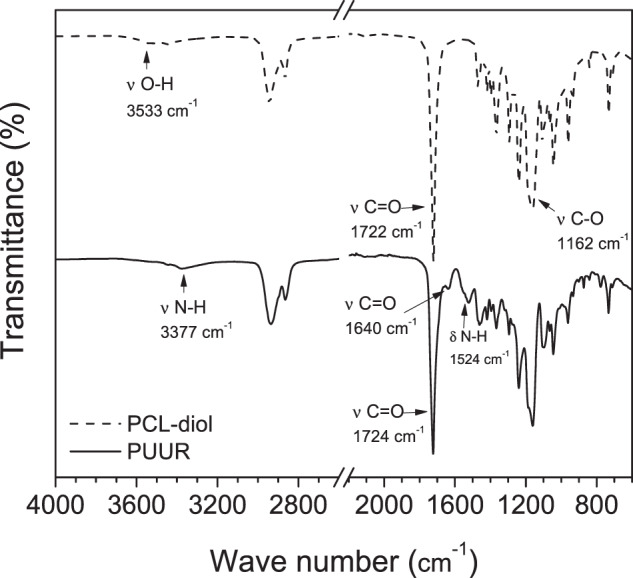
FTIR spectra of PCL-diol and PUUR

The chemical structures of the PUUR and the PCL-diol were confirmed by FTIR and their characteristic signals are presented in Fig. 1. For PUUR, the peak at 3377 cm^−1^ is assigned to the urethane NH group stretching vibrations [29, 30]. The carbonyl stretching (ѵ C=O) is observed at approximately 1724 cm^−1^, both to the –C=O of the ester group of PCL-diol as to the carbonyl of urethane, due to the high proportion of PCL into PUUR [6, 29, 30].

The peak around 1524 cm^−1^ is assigned to amide II adsorption (NH urethane bending vibrations and CN stretch vibrations) [6, 29]. Additionally, a peak at 1640 cm^−1^ is assigned to the urea bonds, confirming the reaction between the amine groups of the extender with the isocyanate groups of the prepolymer (NCO) [29]. No residual isocyanate signal (2275–2263 cm^−1^) was present in PUUR spectrum [6].

Finally, in the PCL-diol spectrum, the signal of stretching of hydroxyl groups (O-H) is observed, which disappears into PUUR due to the formation of the urethane group (NH-COO) [30].

**Fig. 2:**
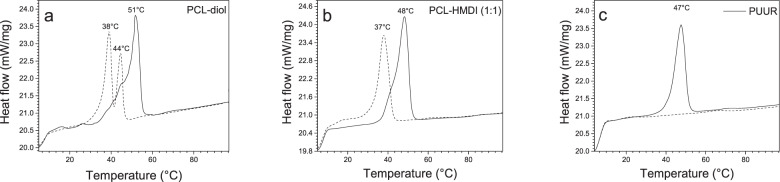
DSC curves for: (**a**) PCL-diol, (**b**) PCL-diol-HDMI (without extender), and (**c**) PUUR. First run (solid line) and second run (dashed line)

The thermal properties obtained by DSC are summarized in Table 3. Regarding PUUR and polyester-urethane formed with PCL-diol and HMDI, it is known that polyurethanes with soft PCL segments exhibit a melting temperature (Tm) around 50 °C [31], a particular sign of PCL-diol (Fig. 2a), which presented two Tm values, characteristics of the multiple morphologies [32].

**Table 3:** Summary of thermal behavior of polymer samples

	Tm (°C)^a^	Tm (°C)^b^
PCL-diol	51	44, 38
PCL-diol - HDMI	48	37
PUUR	47	–

*Tm* Melting temperature

^a^First heating run

^b^Second heating run

In Fig. 2b, c, a decrement in the Tm values of PCL-diol - HMDI and PUUR generated by the formation of covalent bonds and hydrogen bridges in the polymer were observed [33]. On the other hand, the presence of the chain extender in PUUR generates greater restriction in the crystallinity of the polymer. This was observed by the disappearance of the endothermic peak in the second run DSC heating curve, which is attributed to intermolecular interactions in PUUR [33]. Thus, the incorporation of the amino acid as a chain extender in the polyurethane is reconfirmed.

Finally, no fusion of hard segment was detected because the isocyanate used (H_12_MDI) is a mixture of three isomers that substantially inhibits crystallization [34]. In addition, the glass transition temperature (Tg) of the soft segment in PCL-diol 2000 and PCL-diol-HDMI is located from −66 to −56 °C [35] but was not observed due to the test started at 10 °C.

### Membrane characterization

**Fig. 3:**
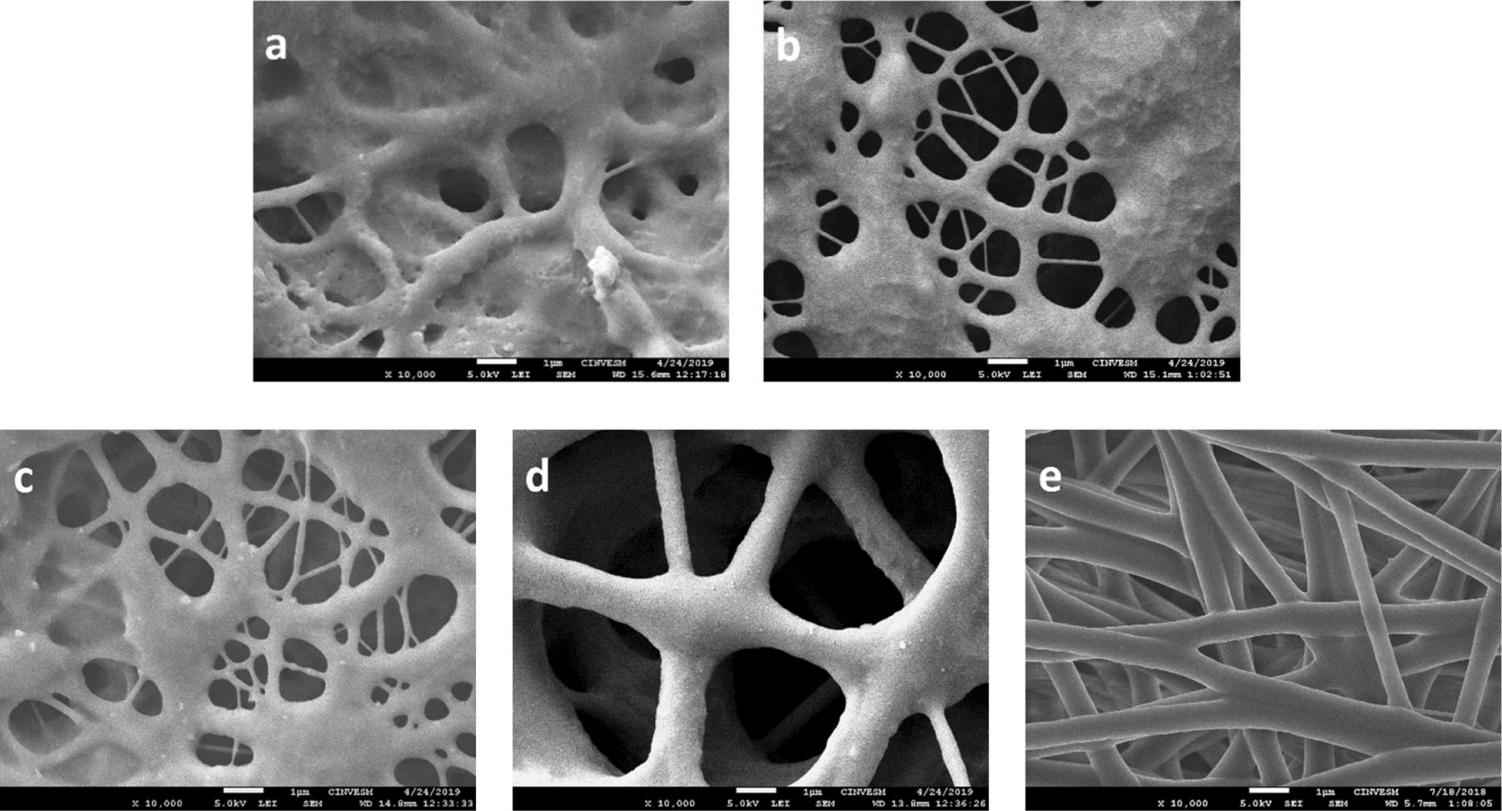
SEM images of the electrospun PUUR membranes (**a**) 20% w/v, 18 kV, 1.2 ml h^−1^; (**b**) 15% w/v, 20 kV, 0.8 ml h^−1^, (**c**) 15% w/v, 20 kV, 1.6 ml h^−1^, (**d**) the PUUE membrane, and (**e**) the Tecoflex membrane

The surface morphology of PUUR, PUUE, and Tecoflex membranes was examined by SEM and their micrographs are shown in Fig. 3. The surface of PUUR membranes (Fig. 3 a–c) exhibited irregular forms as conglomerate fiber noted in PUUR B and C, attributed to excess charges by the voltage used, and flatted fibers in PUUR A as a result of high viscosity of the solution [36]. PUUE membranes obtained at 20% w/v and 10 kV, avoid conglomerates and flat fibers (Fig. 3d).

Regarding the mean fiber diameters (Table 4) of polyurethanes, PUUE sample presented the fibers with largest diameters, due to the higher concentration of the polymer and low voltage used, while PUUR A had bigger fiber diameter than PUUR B and PUUR C samples. With respect to flow rate, it did not show changes in the morphology of the electrospun membrane surfaces.

**Table 4:** Electrospinning conditions and measured parameters

	Polymer concentration (%)	Applied voltage (kV)	Flow rate (ml h^−1^)	Fiber diameter (nm)	Pore diameter (µm)	Contact angle of electrospun membrane (°)	Contact angle of dense membrane (°)
Tecoflex	9	20	1.0	733 ± 51	3.6 ± 0.8	121 ± 0.6	66 ± 1.0
PUUE	20	10	1.2	2166 ± 494	4.0 ± 0.8	147 ± 0.5	73 ± 0.2
PUUR							
A	20	18	1.2	620 ± 99	1.1 ± 0.2	58 ± 0.9	65 ± 0.5
B	15	20	0.8	329 ± 78	1.1 ± 0.2	76 ± 0.2	
C			1.6	323 ± 66	1.0 ± 0.2	113 ± 1.0	

The contact angle results are also described in Table 4. The obtained dense membranes present contact angles between 65° and 73°, confirming the moderately hydrophilic character reported for membranes based on this type of PU [3, 6]. Note that the most hydrophilic polyurethane was the PUUR membrane due to the presence of polar functional groups in its chain, which explains the increment in wettability in comparison with PUUE that is a non-amino acid polyurethane.

For the electrospun membranes, an increase in the contact angle values than the respective dense membranes was observed, despite being the same polymer. This was expected since the presence of roughness by electrospun fibers results in the increase of the contact angle [37]. On the other hand, the processing conditions, chemical structure and microstructure irregularities at the surface as flattened fibers are the factors of contact angle decrease in PUUR A.

### In vitro biological testing of electrospun and dense membranes

#### Material toxicity

**Fig. 4:**
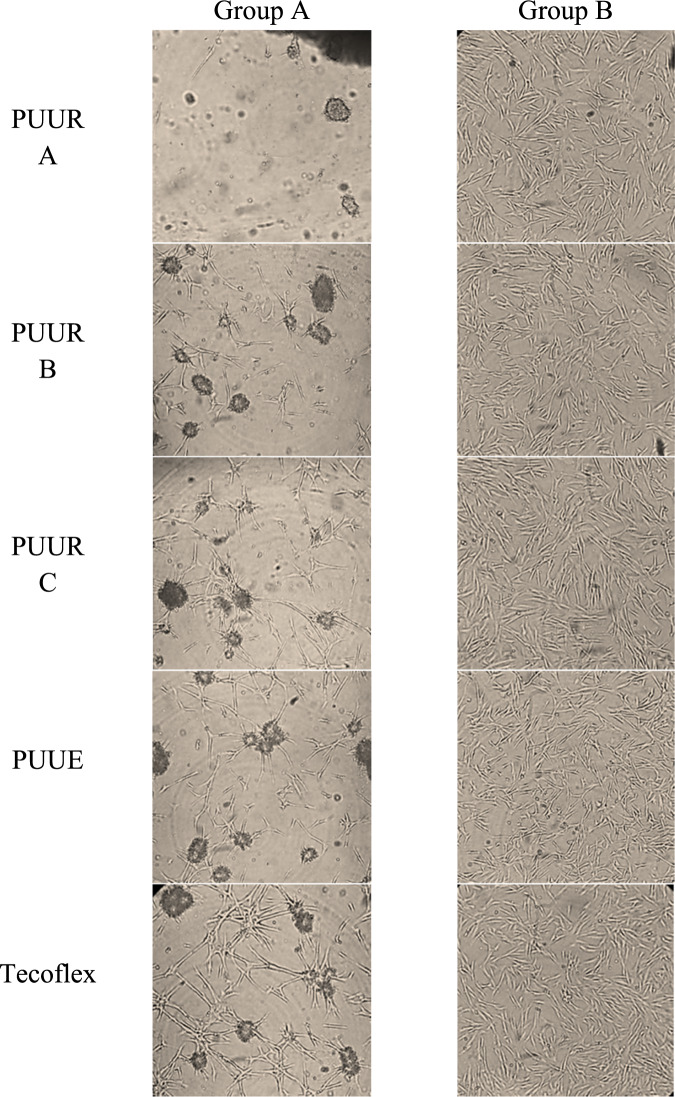
Morphology of hFB cells cultured for 24 h on unwashed (group A) and washed (group B) of electrospun poly(urea-urethane) membranes (PUUR A, PUUR B, PUUR C, and PUUE) and Tecoflex

Optical microscope observation and conventional MTT assay were used to evaluate the toxicity of PUUR, PUUE, and Tecoflex membranes to hFB cells. Figure 4 shows the optical images of cells exposed to two groups of electrospun membranes, group A and B, for unwashed and washed membranes, respectively. In group A, it was observed cell damage (cells with rounded and swollen shapes, and decreased of cell number), and resulting in the development of signs of toxicity. On the contrary, cells in group B retained their typical spindle shape and cell confluence.

**Fig. 5:**
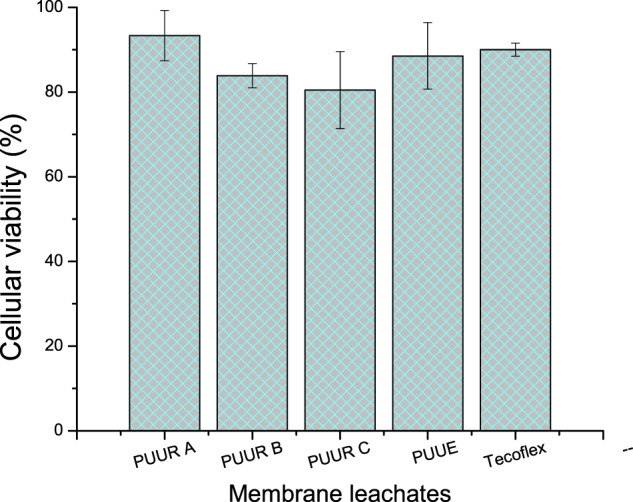
Evaluation of electrospun membranes by comparing the viability of hFB cells in leaching

Regarding the MTT toxicity test (Fig. 5), the leachates obtained from the washed polymers presented %CV values greater than 80%, so that membranes were considered non-toxic based on the ISO 10993-5:2009 standard. Therefore, membranes require washing pretreatment in order to decrease signs of toxicity.

#### Fibroblast cell adhesion

**Fig. 6:**
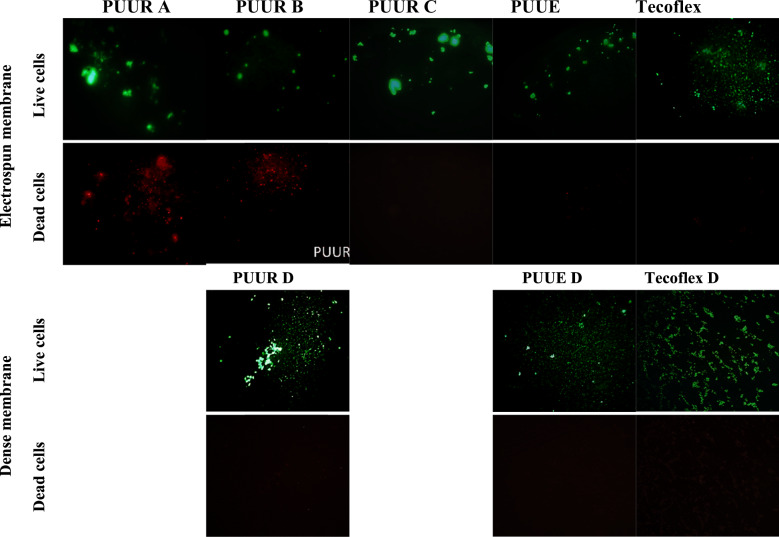
Live/Dead assay of hFB growth on electrospun and dense polyurethane membranes (PUUR A, PUUR B, PUUR C and PUUE) and Tecoflex for 24 h. Green color indicated the active cells whereas red color the dead cells

Figure 6 shows the adhesion of hFB in electrospun and dense membranes after the LIVE/DEAD test, specifically, green fluorescence for viable cells and the red fluorescence for dead cells.

It was observed adhesion of hFB in all membranes. However, clustered areas of green fluorescence were more evident in PUUR membranes than in electrospun and dense membranes from others samples, specifically dense membranes, that showed uniformly scattered cells (green spots).

Regarding to contact angle and hFB adhesion, this type of membrane presented contact angles between 65° and 72° (Table 4), values reported for fibroblast cell adhesion [38]. Although electrospun membranes, the contact angle increased by the morphology, they have adequate wettability to carry out cell adhesion without reducing cell extension on membrane.

**Fig. 7:**
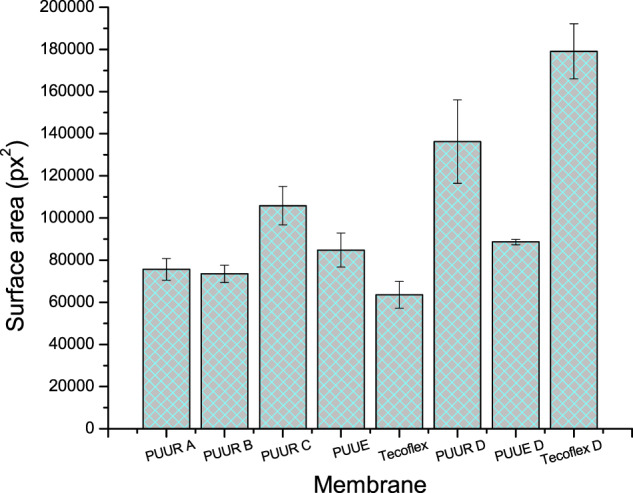
Cell surface area of live hFBs on electrospun and dense membranes

The relationship between fiber diameter with cell adhesion was determined by using the fluorescent surface area (pixel^2^) of live cells from electrospun membranes (Fig. 7), considering that this parameter is not a cell count. From PUUR, despite that PUUR B and C do not differ in composition and had similar fiber diameter, 329 ± 78 and 323 ± 66 nm, respectively, PUUR C presented higher green fluorescent surface area. As can be seen, electrospun PUUE presented the largest diameter (2166 ± 494 nm), and a fluorescent area close to the PUUR A and B membranes. On the contrary, dense membrane of Tecoflex had greater fluorescent surface area than electrospun membranes and dense PUUR and PUUE, being dense PUUR in second place.

Finally, the red fluorescence indicates adhering cells that begin an apoptotic process. It was observed that electrospun materials allow cell adhesion and viability, however, PUUR A and B membranes show cell damage and, therefore, are indicative of toxicity.

#### Cell adhesion and morphology studies by FESEM

**Fig. 8:**
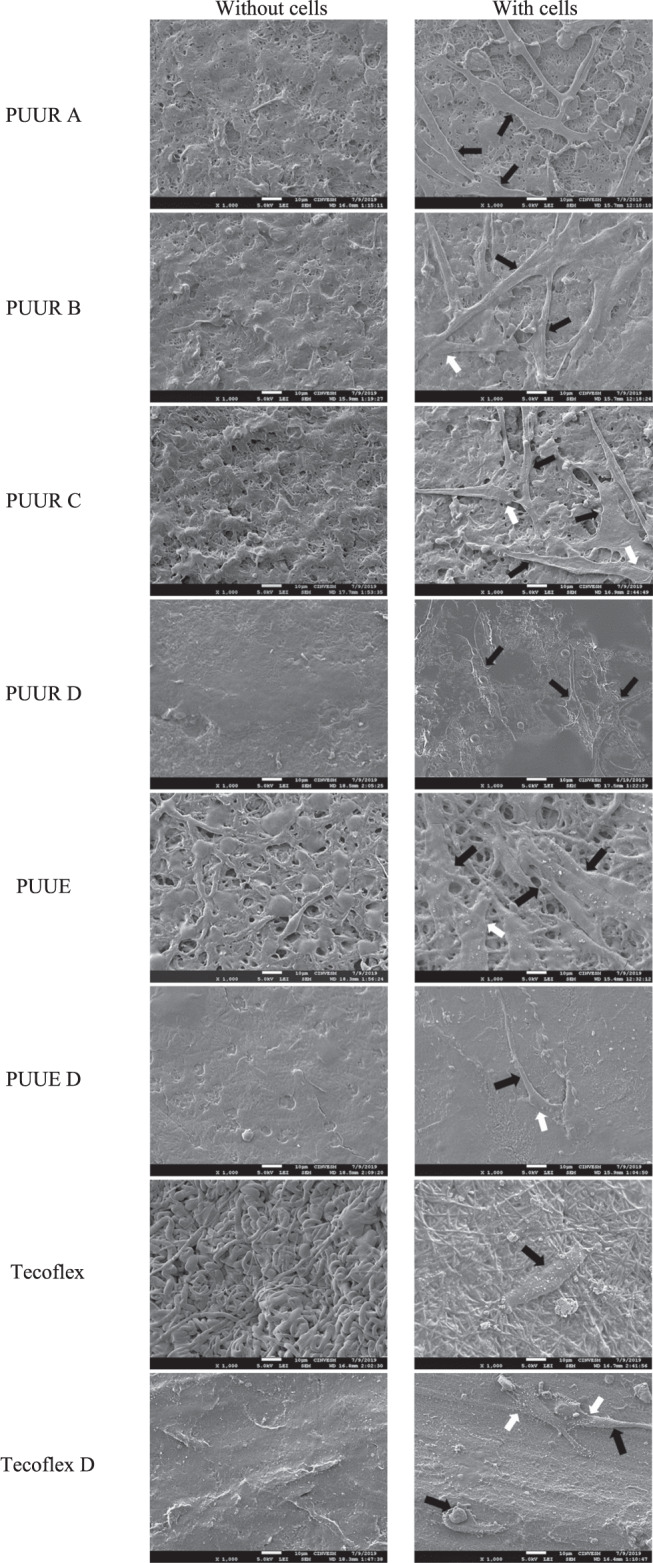
SEM images of hFB cells after 24 h of incubation on electrospun and dense polyurethane membranes (PUUR A, PUUR B, PUUR C, and PUUE), and Tecoflex. Black arrows show cells and white arrows indicate lamellipodia (1000x; 5 kV)

Before describing the cellular morphological characteristics and because treatment with ethanol to dehydrate the cells affect the integrity of the membranes, either by solubility or swelling [39], the appearance of fibers in Fig. 8 without cells differs from the material without the dehydration treatment.

The morphology of the fibroblasts observed in Fig. 8 corresponds mostly to the typical bipolar spindle and multipolar stellate shape, with elongated and extended hFB on the polymer. Cellular locomotion was inferred by the elongation of hFB, extension of lamellipodia (white arrows) [40], and cell-cell interactions by extension of filopodia, characteristic processes of healthy cells [41]. It was only found in dense Tecoflex the presence of small and rounded morphologies of hFB, which indicate poor adhesion to the surface, growth arrest, or initial apoptotic response [38].

By comparing the dense and electrospun membranes of PUUR, PUUE, and Tecoflex, greater adhesion and extension of hFB were observed in electrospun membranes. Therefore, a better adhesion response of hFB was obtained by the presence of polymeric fibers, compared to their dense counterparts.

Electrospun Tecoflex shows surface changes both in absence and presence of cells; in the absence, the fibers show swelling and in the presence, a coating was observed on the surface of the fibers by the ethanol treatment [14]. Respect to electrospun PUUE membranes, a more ordered distribution of hFB was observed than PUUR, which is related to the morphology of fibers. PUUR scaffolds present fiber heterogeneity due to the presence of beads or flat fibers, while PUUE presents uniform fibers; this morphological difference has a cell adhesion and migration impact [13, 42, 43].

Therefore, both fibrous structures as the membrane composition, play an important role in the adhesion and spread of hFB cells on the material.

## Discussion

The effects of chain extender and fiber morphology on the processing conditions and biocompatibility were studied in our research. The material characterization was carried out by using FTIR, DSC, and GPC techniques to verify the feasibility of amino acid inclusion in the polyurethane synthesis. The evaluation of the materials as a cell scaffolding biomaterial for growing and developing of cells on the material was also realized.

For material characterizations, there is evidence that supports the formation of PUUR in the analyzes performed. In GPC, the use of the chain extender basically led to an increase in Mw that eventually couples to the prepolymer chain. The FTIR results of PUUR showed the absence of isocyanate and hydroxyl signals, corresponding to the precursors, and the emergence of urethane and urea peaks, indicating that -NCO groups (H_12_MDI) reacted fully with -OH (in PCL-diol) and -NH (in arginine), confirming the reaction took place. On another hand some formulations of polyurethanes have a complex morphology that depends on the nature of the hard and soft segments and their ratio; the inclusion of the chain extender generated a decrease in PCL-diol crystallization and in the second run Tm peak was not found, this has been shown in other studies of polyurethanes with PCL-diol (2000 g mol^−1^), where covalent linkages and strong interchain interactions through hydrogen bonding restricted crystallization [33, 44].

In membrane characterization by SEM, the voltage in the electrospinning process of PUUR led to deposition of conglomerate fiber due to an unstable jet formation [12] and inadequate polarization of the material. An increase in viscosity by an increase in solution concentration was another parameter that affects the morphology. Flattened fibers were observed, indicating incomplete evaporation of solvent during electrospinning [36]. There are many structural factors that hinder a stable electrospinning process [45], the non-linear behavior of the elastomeric membranes obtained is complicated due to the high deformability, quasi-incompressibility, softening effects, and time-dependent viscoelastic effects [46].

The wettability of the membranes was studied given that influences the interaction of cells with the surface of the polymer [10, 11]. Therefore, it was observed a lower contact angle in PUUR than in PUUE, which is attributed to the arginine chain extender, consisting of amine and carboxylic acid groups. For electrospun polymers has been reported that fibers generate a greater roughness in the material, preventing the penetration of water into the pores of the membrane due to the suspended liquid in a solid-air surface of the fibers [37], causing an increase in contact angle. This does not affirm the hydrophobic character of the material, which changes with time, depending on the composition and morphology of the material.

About the evaluation of membranes, it was important to determine that electrospun membranes were not toxic to hFB cells to ensure their safety use as skin scaffold. Although PUs are considered non-toxic polymers, these ones can present leaching from degradation products and residues from the synthesis process [3] or from solvents from the electrospinning process [47] with a cytotoxic effect. On the other hand, results of FTIR (Fig. 1) and resistance to thermal degradation of PUUR [3], did not detect residues of isocyanates or degradation of the polymer at the test temperature. Therefore, the solvent residue from the electrospinning process is responsible for cytotoxicity. It has been reported that the exposure of fibroblasts to DMF generates cell shedding, changes in morphology [48], and death by apoptosis after 24 h [49]. Vacuum drying of membranes was insufficient to remove all solvent residues. In turn, removal by temperature was difficult due to its boiling point (153 °C) [47] and the melting temperature of the PU membranes in this work (~47 °C); thus, membranes require washing with sterile distilled water prior use.

As previously mentioned, cellular response in terms of adhesion is affected by scaffold properties like surface chemistry and topography. Hence fluorescence staining, and SEM analysis were necessary in the evaluation response. Staining tests demonstrated the presence of clusters, especially in electrospun membranes. This kind of clustering has been reported as the extension of cells adhered to the surface of the material [50] or cells agglomeration with poor interactivity between the substrate and cells [4]. However, the values of contact angles presented in dense membranes were not values reported that promote cells agglomerates [4], and cells with round shapes and aggregated into clusters were not observed in SEM images, except for dense Tecoflex. Therefore, the absence of these morphologies in other membranes corroborates that the fluorescent areas observed in electrospun membranes by staining with the LIVE/DEAD kit are mainly due to the spread of hFB rather than poor interaction with the material. Thus, can be concluded that, although in the previous test there was a greater green fluorescence area in dense Tecoflex membrane, the morphology of the cells does not indicate a high affinity, nor the characteristic form of hFB. Otherwise, for dense PUUR membrane, this effect was observed and is attributed to a better affinity of the cell due to the arginine chain extender. It has been studied in the literature that for hFB cells, the presence of amino groups in the polymer structure cause by amino acid arginine generates greater cellular affinity [15, 17].

For electrospun morphology, although membranes with submicron fiber diameters tend to promote adhesion and cell viability [51], is well know that the presence of agglomerates from electrospinning process impair cell adhesion by providing a smaller focal area of adhesion between the fiber and the cell [17]. However, PUUR membranes exhibited in micrographs (Fig. 8) extended shapes of hFB, which indicates a good interaction between the material and cells. Additionally, it was observed a surface covering in Tecoflex fibers. This can be due to the excretion of extracellular matrix (ECM) components by the fibroblast cell, given that fibrous scaffold structures can cause ECM production [14, 42]. Thus, Tecoflex electrospinning is considered to promote good adhesion and stimulation of the cell. Remember that cells use only temporary contacts with the nano-surface and spend more time searching for the best position to establish themselves and to produce morphogenic factors and inducer molecules [52]. However, no complementary information was obtained to confirm the excretion of components of the extracellular matrix by the cells.

Finally, dead cells were attributed to residual solvent in the electrospun membranes [53] and the effect that this has on the permeation properties, especially in electrospun materials from PUUR A and B. It was observed that cell adhesion and later spread of cells were defined by both the topography and composition of the scaffold surface.

## Conclusions

In the present work, an electrospinning method was implemented for preparing fibrous PU membranes containing arginine as a chain extender. The results showed that the mean diameter of the fibers increased with the increased polymer concentration. Contact angle tests showed that PUUR dense membranes present moderately hydrophilic properties; however, the contact angles in the electrospun membranes do not show a determinant result in adhesion evaluation of hFB. The results of cell culture showed that the fibroblast cells adhered to the membranes, achieving greater elongation and extension of hFB in electrospun membranes than in dense membranes. Fibroblasts grown on PUUR membranes showed a different adhesion pattern, indicating cell mobility in search of the best adhesion site on the fibrous surface. The presence of the amino acid in the composition of the material contributed to the affinity of hFB with the material, given that proliferative morphologies (fibroblast extension) were observed, while Tecoflex dense membranes presented majority non-proliferative morphologies. It is concluded that the presence of fibers has a greater impact on cell/membrane adhesion than dense membranes as observed by SEM images.
